# One-step resonant controlled-phase gate on distant transmon qutrits in different 1D superconducting resonators

**DOI:** 10.1038/srep14541

**Published:** 2015-10-21

**Authors:** Ming Hua, Ming-Jie Tao, Fu-Guo Deng, Gui Lu Long

**Affiliations:** 1 State Key Laboratory of Low-Dimensional Quantum Physics and Department of Physics, Tsinghua University, Beijing 100084, China; 2 Tsinghua National Laboratory for Information Science and Technology, Beijing 100084, China; 3 Collaborative Innovation Center of Quantum Matter, Beijing 100084, China; 4 Department of Physics, Applied Optics Beijing Area Major Laboratory, Beijing Normal University, Beijing 100875, China

## Abstract

We propose a scheme to construct the controlled-phase (c-phase) gate on distant transmon qutrits hosted in different resonators inter-coupled by a connected transmon qutrit. Different from previous works for entanglement generation and information transfer on two distant qubits in a dispersive regime in the similar systems, our gate is constructed in the resonant regime with one step. The numerical simulation shows that the fidelity of our c-phase gate is 99.5% within 86.3 ns. As an interesting application of our c-phase gate, we propose an effective scheme to complete a conventional square lattice of two-dimensional surface code layout for fault-tolerant quantum computing on the distant transmon qutrits. The four-step coupling between the nearest distant transmon qutrits, small coupling strengths of the distant transmon qutrits, and the non-population on the connection transmon qutrit can reduce the interactions among different parts of the layout effectively, which makes the layout be integrated with a large scale in an easier way.

Universal quantum gate is the key element for quantum computation^1^,^2^,^3^,^4^,^5^,^6^,^7^,^8^,^9^. Two-qubit universal controlled-phase (c-phase) gate, the equivalent of two-qubit controlled-not (CNOT) gate (or the hyper-parallel two-photon CNOT gates on photon systems with two degrees of freedom^7^,^8^,^9^), can form universal quantum computing assisted by single-qubit operations, and it has attracted much attention in recent years. To realize the deterministic quantum entangling gates, nonlinear interactions on qubits are required. Cavity quantum electrodynamics (QED)^10^ provides a promising platform to realize the nonlinear interaction between an atom and a field, and it can achieve indirect nonlinear interaction among atoms or fields. To simulate cavity QED, atom^11^,^12^,^13^, spin^14^,^15^,^16^,^17^,^18^,^19^,^20^,^21^,^22^,^23^,^24^,^25^, or superconducting qubits^26^,^27^,^28^,^29^,^30^,^31^,^32^,^33^,^34^,^35^,^36^ coupled to optical cavities^37^,^38^,^39^,^40^,^41^, superconducting resonators^42^,^43^,^44^,^45^, or nanomechanical resonators^46^,^47^ have been studied a lot for quantum information processing both in experiment and in theory^48^.

Circuit QED, composed of a superconducting qubit coupled to a superconducting resonator^42^,^43^, gives a powerful candidate platform for quantum computation^49^ because of large-scale integration of superconducting qubits and all-electrical control using standard microwave and radio-frequency engineering techniques. It can work from the dispersive weak regime to the resonant strong regime^50^, and even the ultra-strong regime^51^. In microprocessors based on circuit QED, there are some interesting types of integration of superconducting qubits or resonators for quantum information processing, including several qubits coupled to a resonator^52^,^53^,^54^, several resonators coupled to a qubit or several qubits^55^,^56^,^57^,^58^,^59^,^60^,^61^,^62^,^63^,^64^, or some circuit QED systems coupled to each other by using qubits, superconducting transmission lines, or capacitance^65^,^66^,^67^,^68^,^69^. The basic tasks of quantum computation in circuit QED have been demonstrated in experiment, such as the c-phase gate^52^,^70^,^71^,^72^ and the controlled-controlled-phase gate^53^,^54^ on transmon qubits in the processor by integrating several superconducting qubits coupled to a 1D superconducting resonator, the generation of the entangled states on transmon qubits^73^ or two resonator qudits^60^, and the measurement on superconducting qubits^69^,^74^ or the microwave photons in a superconducting resonator^75^,^76^,^77^,^78^.

To avoid the indirect interaction among qubits in the processor by integrating more superconducting qubits coupled to a 1D superconducting resonator for complex quantum computation, one should take much smaller coupling strength between a qubit and the resonator or tunable coupling qubits. To integrate more resonators coupled to a qubit, smaller or tunable coupling between the qubit and each resonator is required as well. Small coupling strength leads to a slow quantum operation which limits the performance of the quantum computation due to the coherence time of qubits and decay rate of resonators. Tunable coupling between a qubit and multiple resonators increases the difficulty to design the superconducting circuits. As another candidate for integration of large-scale quantum computation, superconducting qubits hosted in different resonators interconnected by a qubit has been studied in experimental and theoretic works^67^,^71^. Up to now, there are no schemes to construct the multi-qubit universal gates on the distant transmon qubits in the similar systems.

In this paper, we propose a scheme to complete the c-phase gate on two distant transmon qutrits (DTQs) hosted in different resonators interconnected by a connection transmon qutrit (CTQ). Different with the schemes for entanglement generation and information transfer in the similar device^67^, our c-phase gate on two DTQs is achieved with one step by taking the same frequencies of qutrits and resonators and small coupling strengths of DTQs. Finally, we discuss the feasibility about its possible experiment implementation with the similar systems in previous works^70^,^71^ and construct a conventional two-dimensional surface code (SC) layout^79^,^80^ as an interesting possible application of our c-phase gate. Although our layout needs extra CTQs than the one in the previous work^70^, there is almost no demand on the life time of the CTQ as the information does not be populated in it during the gate operation, and the interactions between nearest DTQs are reduced into four-step coupling. On one hand, the small coupling strength of DTQs can reduce the interactions between a qutrit and the nearest resonators. On the other hand, four-step coupling between nearest DTQs can be turned on and off easily by CTQs. These characters make our layout suitable to be integrated with a large scale.

## Results

### C-phase gate on distant transmon qutrits

**Figure 1:**
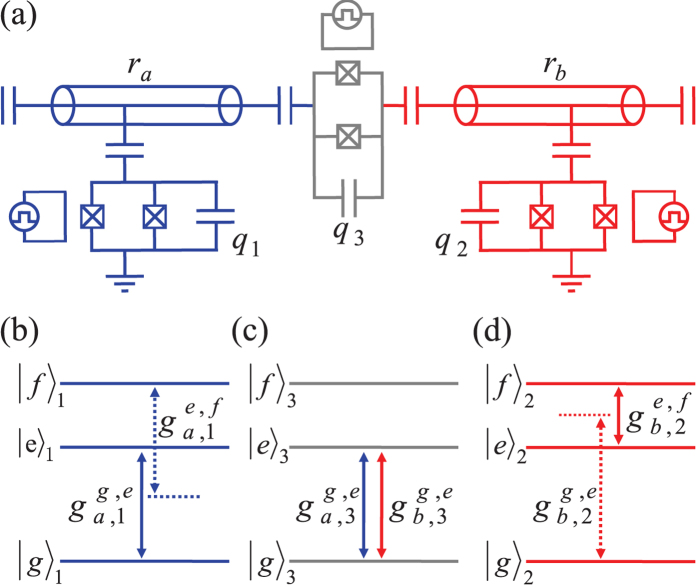
(**a**) The setup for the construction of our c-phase gate on the distant transmon qutrits *q*_1_ and *q*_2_. *q*_1_ (*q*_2_) is coupled to the high-quality resonator *r*_*a*_ (*r*_*b*_). The two resonators are interconnected by a connection transmon qutrit *q*_3_. (**b**–**d**) are the illustrations of interactions between *q*_1_ and *r*_*a*_, *q*_3_ and *r*_*a*_ (*r*_*b*_), and *q*_2_ and *r*_*b*_, respectively.

Let us consider a system composed of two DTQs coupled to different superconducting resonators interconnected by a CTQ, shown in Fig. 1. The Hamiltonian of the system in the interaction picture is (*ħ* = 1)


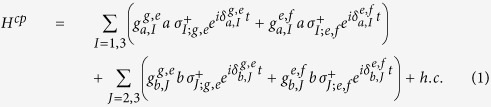


Here, *a* and *b* are the annihilation operators of the resonators *r*_*a*_ and *r*_*b*_, respectively. 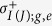 and 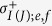 are the creation operators of the transitions 
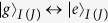
 and 
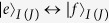
 of *q*_*I*(*J*)_, respectively. 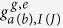 and 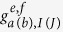 are the coupling strengths between the two transitions of *q*_*I*(*J*)_ and *r*_*a*(*b*)_, respectively. 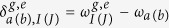 and 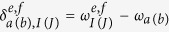. 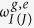 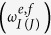 is the transition frequency between the states 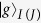 and 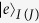 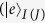 and 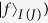 of the qutrit *q*_*I*(*J*)_. *ω*_*a*(*b*)_ is the frequency of the resonator *r*_*a*(*b*)_. |*g*〉 is the ground state of a transmon qutrit, and |*e*〉 and |*f* 〉 are the first and the second excited states, respectively.

In order to obtain the effective Hamiltonian of the system composed of the two resonators (*r*_*a*_ and *r*_*b*_) and three superconducting qutrits (*q*_1_, *q*_2_, and *q*_3_) to construct our c-phase gate, we take small values of 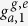, 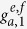, 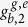, and 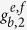 with 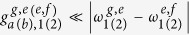, and 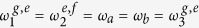 to make the transitions 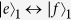 of *q*_1_ and 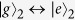 of *q*_2_ to detune largely with *r*_*a*_ and *r*_*b*_, respectively, which indicates the dispersive coupling between the transition 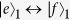 and *r*_*a*_ and that between 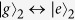 and *r*_*b*_ can be ignored. Besides, only the transition 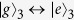 of the CTQ *q*_3_ should be considered as the coupling is the dispersive one and there is just one microwave photon can be generated in the resonators (just the transitions 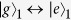 of *q*_1_ and 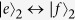 of *q*_2_ are used in our scheme), respectively. Here, the Hamiltonian can be reduced from Eq. (1) to


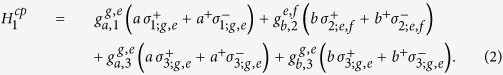


To our purpose, we then take the transformations 
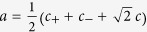
, 
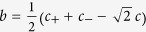
, and 
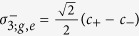
 with the condition 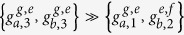 and 
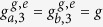
81,82,83. The transformations give us three new normal modes and only one of them (that is, *c*) resonates with the qutrits, so we can ignore the other two detuning modes and the system is reduced to a two-qubit one resonantly coupled to a single mode of the resonant field (further details can be found in the method). Eq. (2) becomes


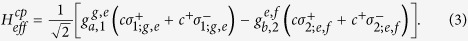


Here, the frequencies of *c* mode and *c*_±_ mode are *ω*_*a*(*b*)_ and 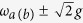, respectively, and the modes of *c*_±_ are highly suppressed, which indicates the information cannot be populated in the state 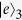 of *q*_3_. Here *c*, *c*_−_, and *c*_+_ are three normal composite-particle operators.

If we take the initial states of the system with the Hamiltonian 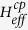 are 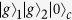, 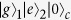, 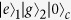, and 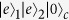, respectively, the evolutions of the system can be expressed as


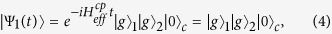



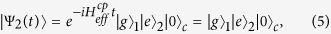



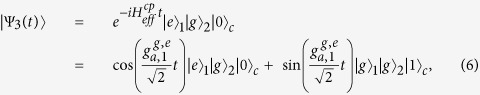



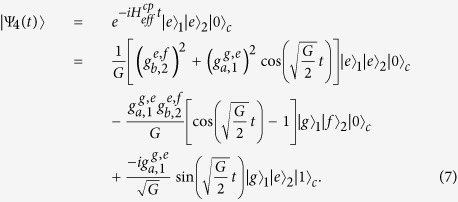


Here 
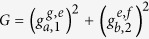
. By using these evolutions, we can construct the c-phase gate on *q*_1_ and *q*_2_. Its principle can be described as follows.

Suppose that the initial state of the system shown in Fig. 1 with the Hamiltonian 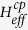 is


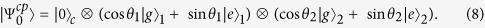


Here 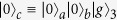. By evolving the system with 
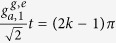
 and 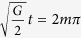 (*k*, *m* = 1, 2, 3, . . .), one can keep the states 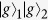 and 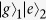 unchanged from Eqs. (4) and (5), respectively. Meanwhile, the state 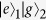 undergoes an odd number of periods and generates a minus phase from Eq. (6), and the state 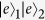 goes through an even number of periods and maintains unchanged from Eq. (7). That is, the system evolves from Eq. (8) into


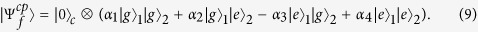


Here, *α*_1_ = cos *θ*_1_cos *θ*_2_, *α*_2_ = cos *θ*_1_sin *θ*_2_, *α*_3_ = sin *θ*_1_cos *θ*_2_, and *α*_4_ = sin *θ*_1_sin *θ*_2_. This is just the result of a c-phase gate on *q*_1_ and *q*_2_, whose matrix reads


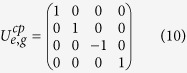


in the basis 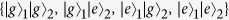.

### Possible experimental implementation and the SC layout

#### The performance of our c-phase gate

To show the fidelity of our c-phase gate on the two distant qutrits *q*_1_ and *q*_2_, we numerically simulate the fidelity of our c-phase gate with the Hamiltonian *H*^*cp*^ of the whole system which contains the following dispersive couplings:


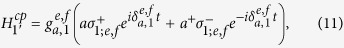



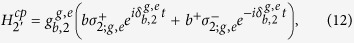



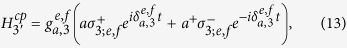



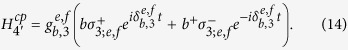


The dynamics of the system is determined by the master equation


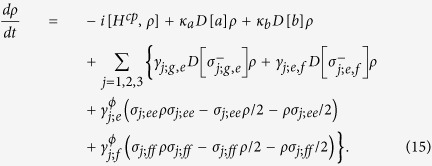


Here, *κ*_*a*,*b*_ is the decay rate of the resonator *r*_*a*,*b*_. *γ*_*j*;*g*,*e*_ (*γ*_*j*;*e*,*f*_) and 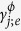 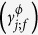 are the energy relaxation and the dephase rates of the transition 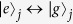 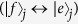 of *q*_*j*_, respectively. 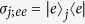 and 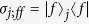. *D*[*L*]*ρ* = (2*LρL*^+^ − *L*^+^*Lρ* − *ρL*^+^*L*)/2.

Let us define the fidelity of our c-phase gate as^17^,^58^,^81^


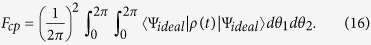


Here 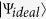 is the final state of a system by using an ideal c-phase gate operation on its initial state 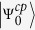 with the effective Hamiltonian 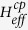. *ρ*(*t*) is the realistic density operator after our c-phase gate operation on the initial state 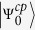 with the realistic Hamiltonian *H*^*cp*^ in which the coherence time of qubits, decay rates of resonators, and the unwanted influence on qutrits from the unresonant parts should be taken into account. By taking the feasible experimental parameters as 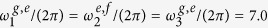 GHz, 
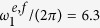
 GHz, 
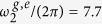
 GHz, 
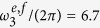
 GHz^84^, *ω*_*a*_/(2*π*) = *ω*_*b*_/(2*π*) = 7.0 GHz, 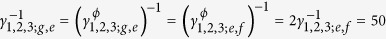 *μ*s^85^, 
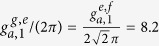
  MHz, 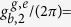

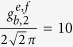
 MHz, 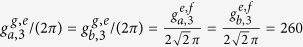 MHz^71^, and *κ*_*a*_ = *κ*_*b*_ 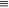 *κ* = 50 *μ*s^44^, we numerically simulate the fidelity of our c-phase gate, which can reach 99.5% within 86.3 ns. Here the coupling strengths of *q*_1_ and *q*_2_ are the optimized ones with *k* = *m* = 1.

**Figure 2:**
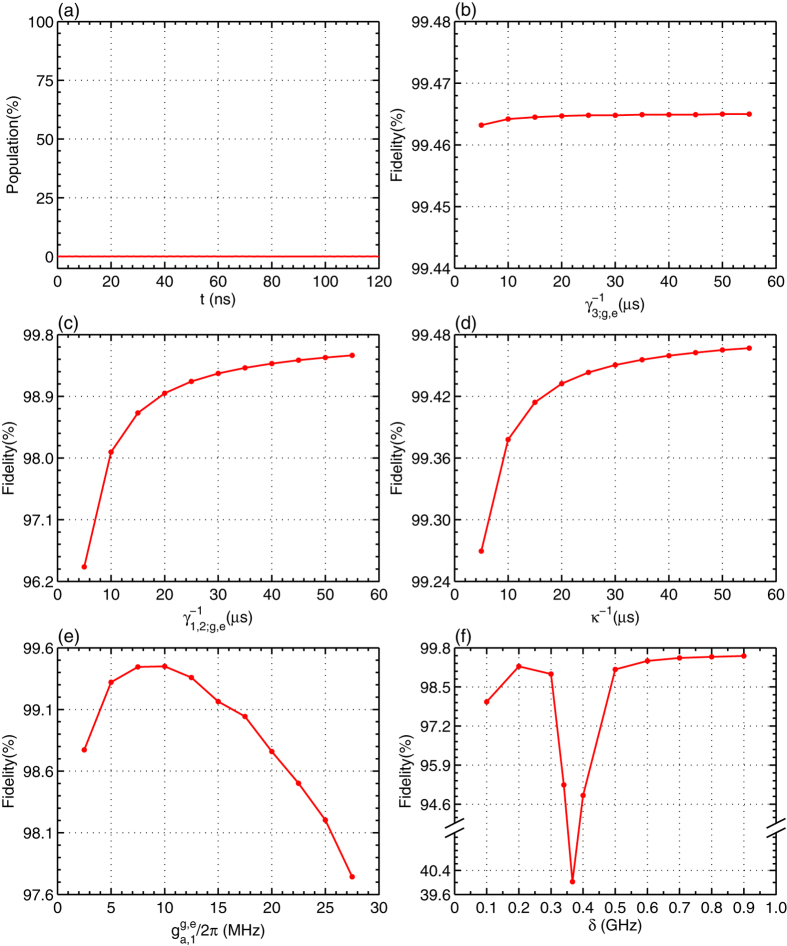
(**a**) The probability of the information populated in the state 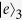 of *q*_3_ 
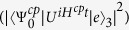
 during the c-phase gate operation on the maximally entangled state of *q*_1_ and *q*_2_ with 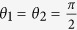 in Eq. (8). (**b**–**f**) The fidelity of the c-phase gate on the DTQs *q*_1_ and *q*_2_ varies with 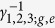, *κ*, 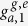, and the anharmonicity of the two transitions of *q*_1_ and *q*_2_ 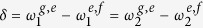.

To show the possible influences from the realistic condition, we give the relation between the fidelity of our c-phase gate and one of the parameters *γ*_1,2,3;*g*,*e*_, *κ*, 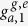, and 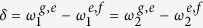, shown in Fig. 2. In Fig. 2(a), the probability of the information populated in the excited state of the CTQ is almost zero, which indicates the assumption that we take *q*_3_ as a two-energy-level qubit for obtaining Eq. (2) is reasonable. This agrees with the relation between the fidelity and the energy relaxation rate *γ*_3;*g*,*e*_ of *q*_3_, shown in Fig. 2(b). Figure 2(c–f) show that the fidelity of the c-phase gate can be enhanced by a longer life time, small coupling strengths, and large anharmonicities *δ* of *q*_1_ and *q*_2_. In Fig. 2(e), the fidelity of the gate is enhanced when 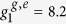 MHz (the operation time of the gate is about 86.3 ns), compared with the one when 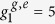 MHz (the time is about 140.5 ns). In Fig. 2(f), the fidelity of our c-phase gate is reduced largely when the anharmonicity of the CTQs is *δ* = 0.37 GHz as the transition 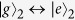 of *q*_2_ is resonant with the mode *c*_+_ at this time, which leads to the influence that its excitation cannot be suppressed. In detail, the difference between the effective Hamiltonian 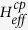 and the realistic Hamiltonian *H*^*cp*^ becomes a large one. The overlape between the final states 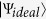 and *ρ*(*t*) obtained by the evolutions with 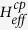 and *H*^*cp*^, respectively from the same initial state 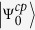 is reduced largely.

#### Application of our c-phase gate in surface code layout

Operations on superconducting qubit cannot perform sufficiently well to let the qubit act as a computational qubit directly with recent techniques and several works are focused on the realization of the surface code on superconducting qubits for fault-tolerant quantum computing. The tolerance of the SC layout to errors allows as high as about 1% error rate of per operation, which is much bigger than 2 × 10^−5^ error rate of the per operation required in quantum correction code^80^. Qubits in SC code are divided into three types: data qubits, measure-z qubits, and measure-x qubits. Away from the boundaries, each data (measure) qubits interact with four measure (data) qubits. As an application of our c-phase gate on two distant transmon qutrits, we construct a SC layout with a conventional square lattice^70^,^80^ for fault-tolerant quantum computing on the DTQs in an effective way.

**Figure 3:**
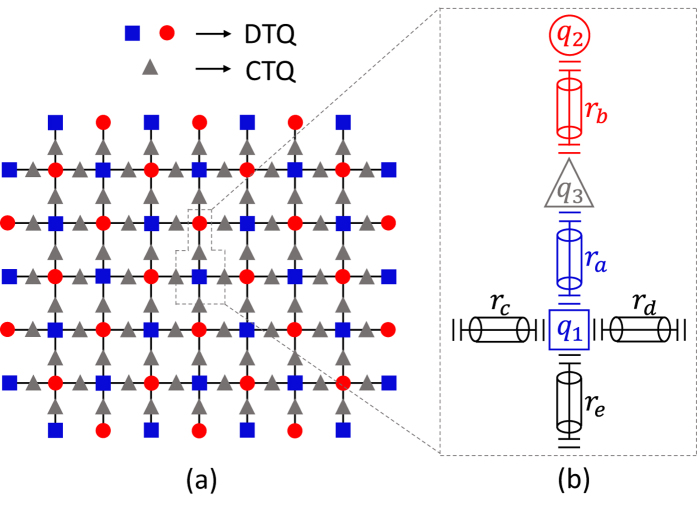
An application of our c-phase gate on two distant transmon qutrits for fault-tolerant quantum computing. (**a**) The setup for the surface code layout. (**b**) A cell of the layout.

Our setup for the SC layout is shown in Fig. 3(a) in which each blue square represents a transmon qutrit with the 6.2 MHz coupling strength, each red circle is a transmon qutrit with the 5 MHz coupling strength, and each gray triangle means a CTQ. The small strengths are used to avoid the interactions between the idle resonators and DTQs. To discuss the performance of our c-phase gate on nearest DTQs in the layout, we consider a cell of the layout shown in Fig. 3(b). Here, *q*_1_, *r*_*a*_, *q*_3_, *r*_*b*_, and *q*_2_ are the same as those in our c-phase gate shown in Fig. 1. The couplings between *q*_1_ and *r*_*c*_, *r*_*d*_, and *r*_*e*_ are considered when the interactions between *q*_1_ and the nearest DTQs are tuned off except for *q*_2_. The Hamiltonian of the cell is


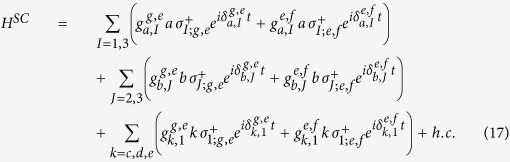


Here, the frequencies of *r*_*c*_, *r*_*d*_, and *r*_*e*_ are taken as 7.5 GHz, 8.0 GHz, and 8.5 GHz, respectively. Except for the coupling strengths which are chosen here as 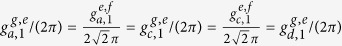



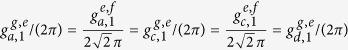



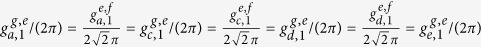

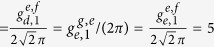
 MHz and 
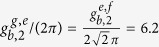
 MHz, the other parameters are the same as the ones in the construction of our c-phase gate. For simplification, we calculate the fidelity of a cell or our c-phase gate on an initial maximally entangled state as


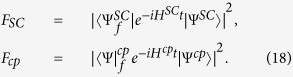


Here


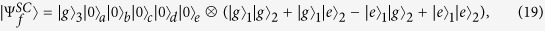



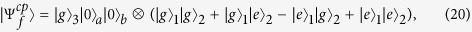



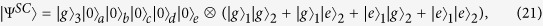



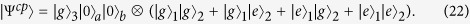


**Figure 4:**
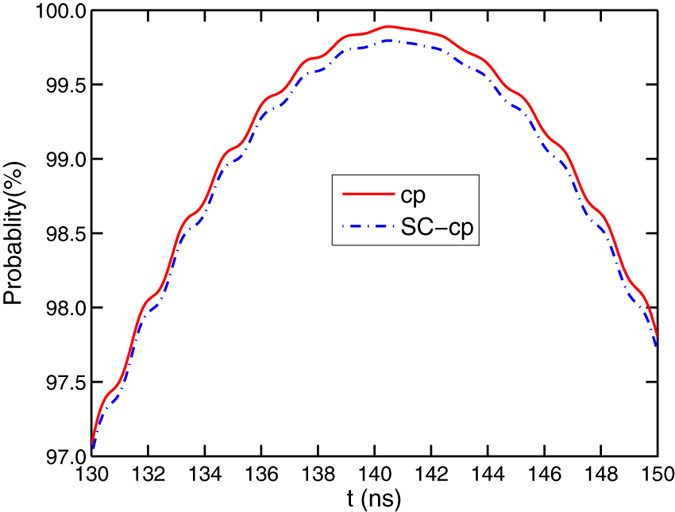
The fidelity of a cell in the surface code layout with our c-phase gate on an initial maximally entangled state of the system composed of *q*_1_ and *q*_2_, 
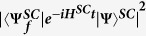
, shown with the blue dash-dotted line. For comparison, the fidelity of our c-phase gate on the same initial state 
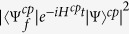
 is given with the red solid line.

The fidelities of both a cell and our c-phase gate on the given initial states change with the time *t*, shown in Fig. 4 in which we do not consider the decay and the energy relaxation rates of the resonators and the qutrits. One can see that the fidelity of a cell composed of our gate and three additional resonators on the given initial state decreases just a little, compared to that of our c-phase gate. Besides, small coupling strengths of DTQs, a tunable range of 2.5 GHz of a transmon qubit^86^, and a tunable range of 500 MHz within 1 ns^87^ of 1D superconducting resonator allow us to maintain the states of the idle qutrits. That is, our c-phase gate works effectively in the construction of the SC layout for fault-tolerant quantum computing.

In the SC layout for fault-tolerant quantum computing^70^,^80^, only the c-phase gate on nearest DTQs are required. Our scheme for the SC layout has some interesting advantages. First, small coupling strengths of DTQs allow us to complete the c-phase gates on nearest DTQs effectively. It can avoid the unwanted interactions from the other transmon qutrits and resonators by choosing proper frequency anharmonicity between a DTQ and its four nearest resonators. Second, CTQs makes the coupling between a pair of DTQs as a four-step one and it can be turned on and off easily. Third, CTQ cannot be excited during the operation of the c-phase gate, and the energy relaxation time of the CTQ has little influence on the fidelity of the gate, which means the tunable-coupling phase qubit with the energy relaxation time of about 130 ns^88^ can also be used here (100 MHz ≫ {6.2, 5.0} MHz). A tunable regime from 0 MHz to 100 MHz^88^ gives us another way to turn on and off the unwanted interactions from the other DTQs in the layout robustly. All these features make the integration of the layout with a large scale easier.

## Conclusion

In conclusion, we have proposed a scheme to construct the c-phase gate on two distant transmon qutrits (*q*_1_ and *q*_2_) which are coupled to different high-quality 1D superconducting resonators (*r*_*a*_ and *r*_*b*_) intercoupled by a CTQ (*q*_3_) in the resonant regime of 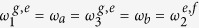. The gate on distant transmon qutrits has not been studied before. Maybe our scheme can support the solid-state quantum computation based on this device. With our c-phase gate, we have proposed a SC layout for fault-tolerant quantum computing on transmon qutrits, which has attracted much attention^70^,^72^ as the error rate of quantum gate is hard to be reduced to 10^−5^ with recent techniques. The layout can be devided effectively into some cells by tuning the frequency of CTQs to detune with two nearest resonators largly. It can avoid the interactions from the other parts of the layout and provides a probability for the large scale integration of a SC layout for fault-tolerant quantum computing with circuit QED.

## Methods

### Hamiltonian and canonical transformations

In the Schrödinger picture, Eq. (2) can be rewritten as


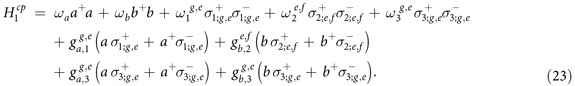


Taking the canonical transformations 
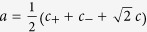
, 
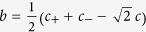
, and 
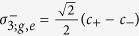
 with the conditions 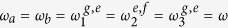 and 
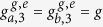
, the Hamiltonian in Eq. (23) can be expressed as


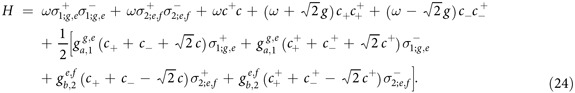


The frequencies of modes *c*_±_ are 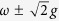. When we take 
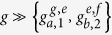
, the excitations of modes *c*_±_ are highly suppressed as it detunes with the resonance modes (*c*, *q*_1_, and *q*_2_ with the frequency of *ω*) largely, and the Hamiltonian in Eq. (24) can be reduced into


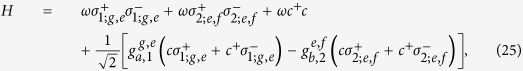


which can be written as


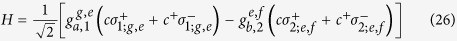


in the interaction picture.

## Additional Information

**How to cite this article**: Hua, M. *et al*. One-step resonant controlled-phase gate on distant transmon qutrits in different 1D superconducting resonators. *Sci. Rep*. **5**, 14541; doi: 10.1038/srep14541 (2015).
